# Metastatic adenocarcinoma of the oesophagus to the submandibular gland—the imaging and pathological features of a rare phenomenon

**DOI:** 10.1259/bjrcr.20160127

**Published:** 2017-03-10

**Authors:** Geoffrey Lie, Jonathan Chia, Jose Quiroga, David Howlett, Jane Topple

**Affiliations:** ^1^Department of Clinical Radiology, Eastbourne District General Hospital, Eastbourne, UK; ^2^Department of Histopathology, Eastbourne District General Hospital, Eastbourne, UK; ^3^Department of Clinical Radiology, Conquest District General Hospital, Saint Leonards-On-Sea, UK

## Abstract

Metastatic disease affecting the submandibular gland is a rare entity, with just over a 100 cases documented. We present the case of a 55-year-old female with an apparently operable oesophageal primary adenocarcinoma, who was identified on staging PET scan and confirmed on ultrasound-guided biopsy to have metastatic disease in her left submandibular gland. The imaging and pathological findings are presented and compared with the current body of literature. Previously recognized primary sites of metastasis include the breast, thyroid, lung and genitourinary organs. We believe this to be the first documented case of oesophageal adenocarcinoma metastasising to the submandibular gland.

## Case

A 55-year-old female presented to gastroenterology outpatients clinic with a 2-month history of dysphagia for solids. Her medical history included learning difficulties, but she was otherwise fit and well.

At upper gastrointestinal endoscopy there was an impassable stenosis 30 cm from the incisors. Biopsies were obtained and revealed a moderately differentiated oesophageal adenocarcinoma. Oesophageal stenting was performed for symptomatic relief. There were no demonstrable metastases on her initial staging CT chest and abdomen, but before proceeding with surgical management a PET scan was arranged.

**Figure 1.**
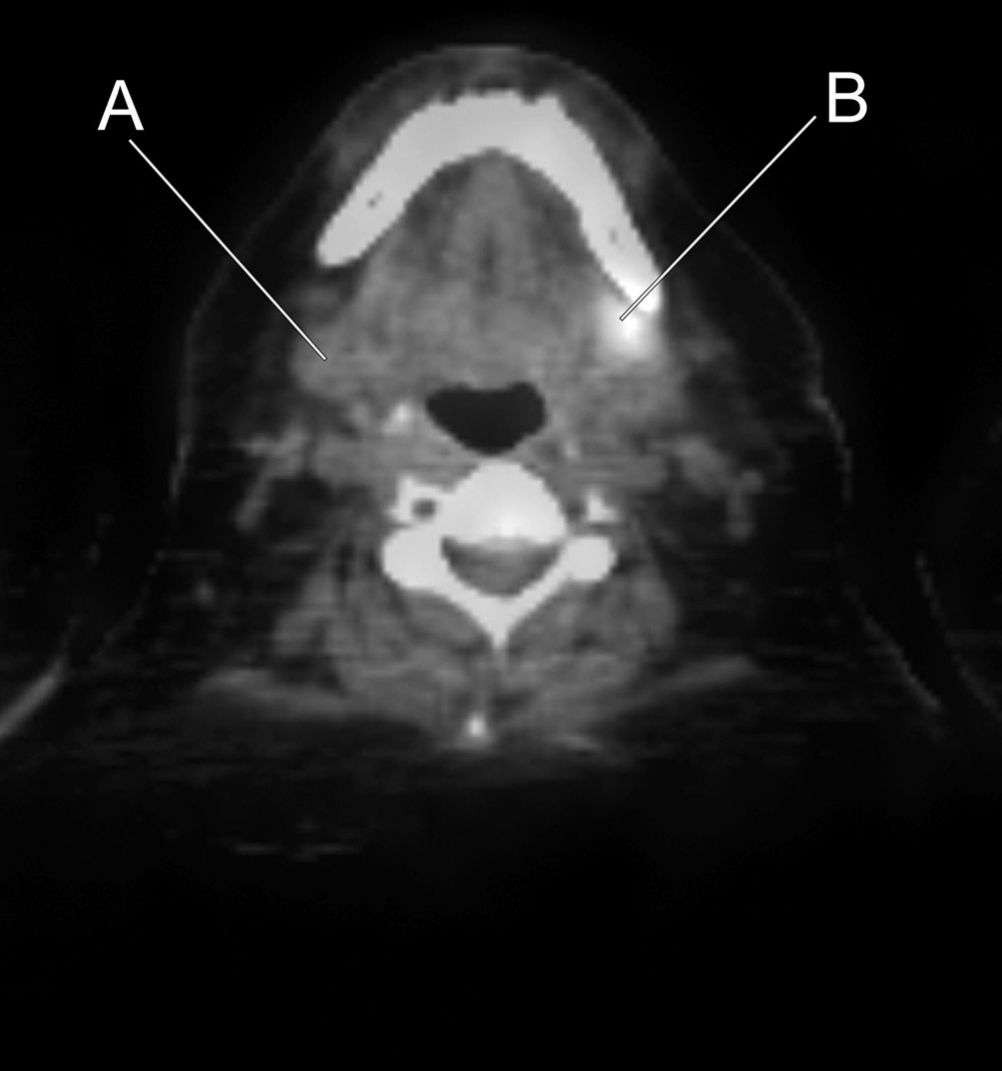
Fused ^18^Fluoro-Deoxy-glucose axial PET CT image demonstrates increased uptake in the left submandibular gland (B). The normal right submandibular gland is shown at this level (A).

**Figure 2.**
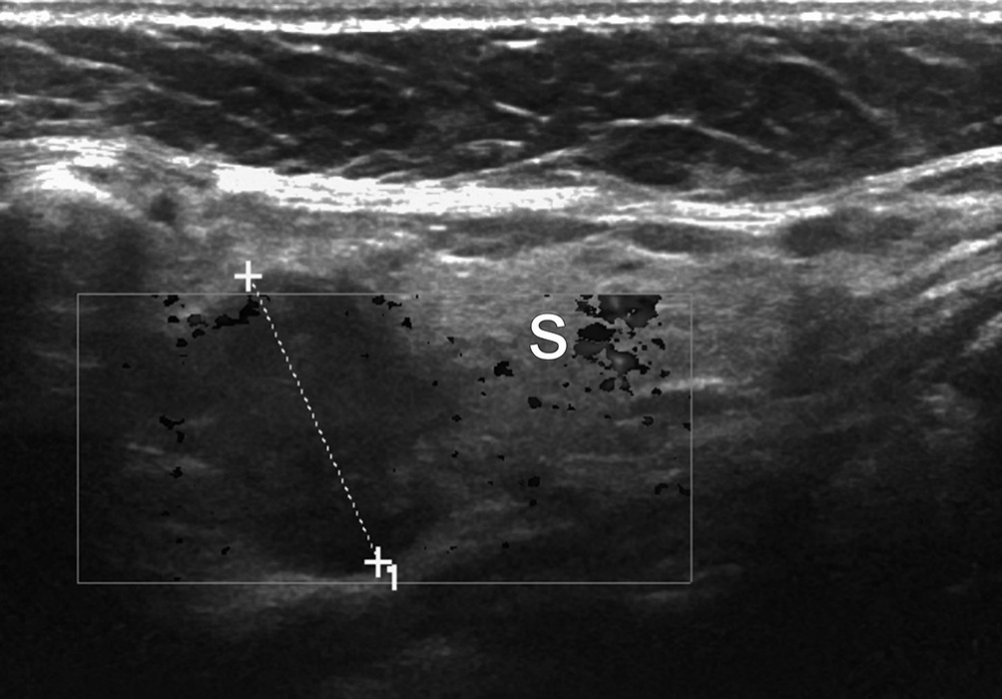
Colour Doppler ultrasound image corresponding to the FDG-avid region on PET-CT. Ill-defined hypoechoic mass within the medial left submandibular gland (calipers), avascular and heterogeneous features those of malignancy though non-specific in differentiating primary from secondary neoplasm.

This detected FDG avid uptake in the left submandibular gland (Figure 1). An ultrasound scan confirmed a poorly defined mass within the left submandibular gland suspicious for malignancy (Figure 2).

**Figure 3.**
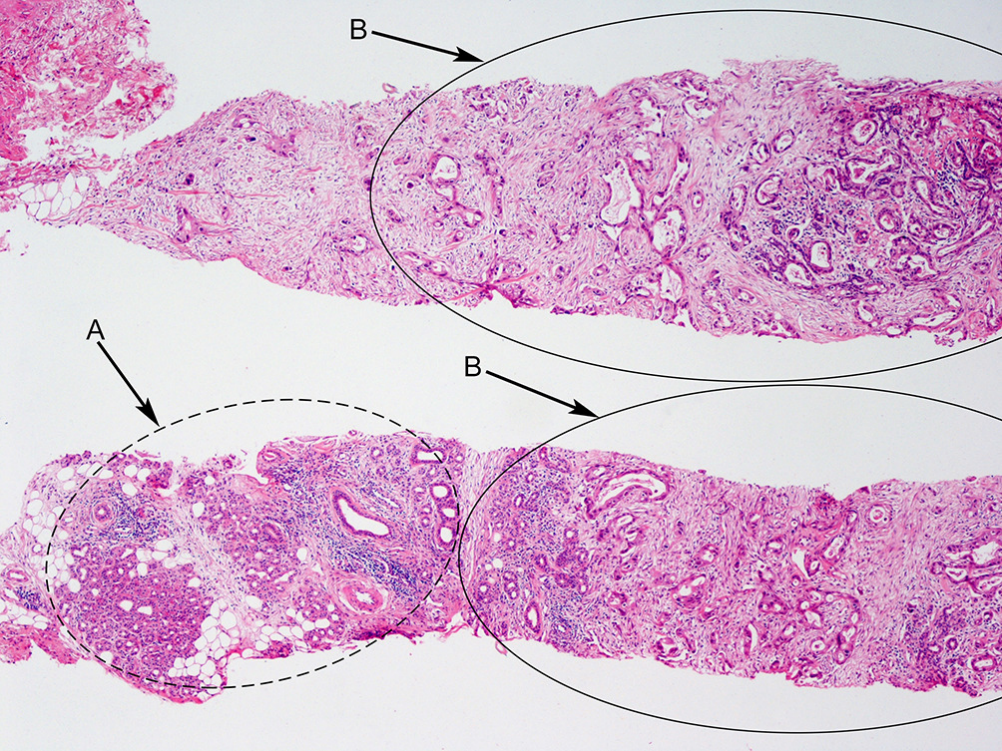
Left submandibular gland, H-E, ×40.

**Figure 4.**
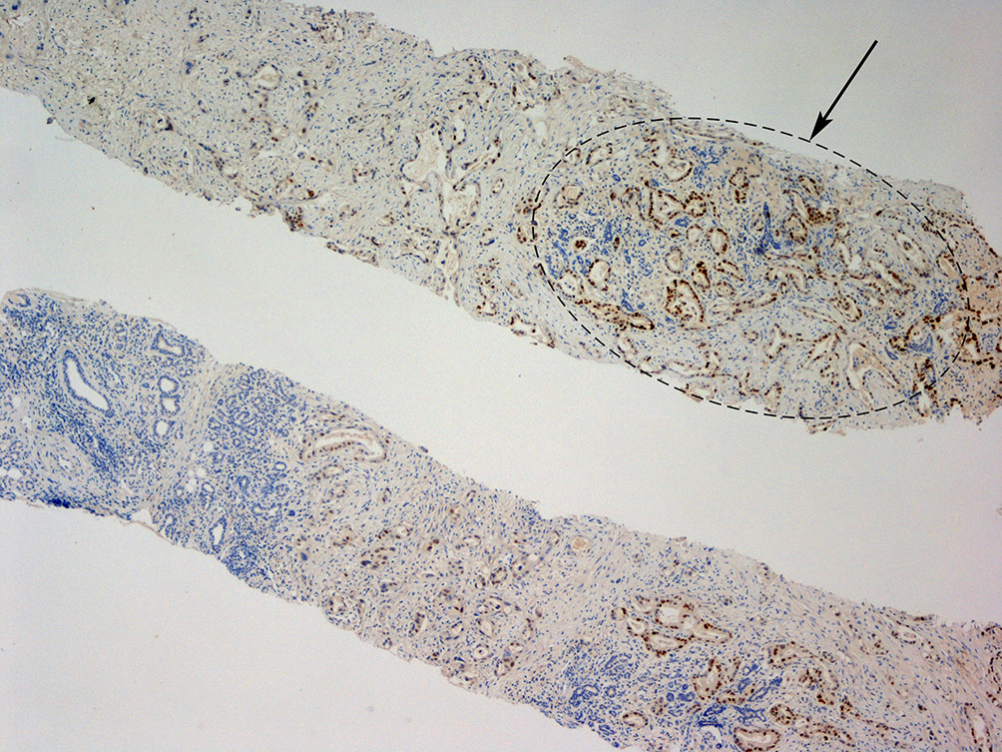
Left submandibular gland, CDX-2 immunostain, ×40. Core biopsies showing normal salivary gland tissue (Figure 3, arrowed a), and infiltration by a proliferation of irregular glands containing intraluminal mucinous secretion and surrounded by desmoplastic stroma (Figure 3, arrowed b). The tumour cells show diffuse nuclear immunoreaction for CDX-2, which indicates intestinal phenotype shown in the Figure 4, arrowed. The immunohistological features are compatible with metastasis of known moderately differentiated invasive tubular adenocarcinoma of the gastro-oesophageal junction.

Two cream-coloured cores were obtained with an 18-gauge needle under ultrasound guidance. Microscopic appearances (Figures 3 and 4) were compatible with metastatic infiltration of the left submandibular gland from the known moderately differentiated oesophageal adenocarcinoma (CK7 and CDX-2 positive/CK-20 negative on immunohistochemistry). The patient was subsequently referred for palliative chemotherapy.

## Discussion

Salivary gland malignancies are uncommon, accounting for 0.4% of all cancers.^1^ Just 3% of salivary gland malignancies are metastatic in origin, the majority of which spread to the parotid gland.^2^ Metastatic disease to the submandibular gland, however, is rare.^3^

The first case of a metastasis to the submandibular gland was reported by Grage and Lober in 1962.^4^ Metastases typically arise from remote sites—often infraclavicular in origin—and spread haematogenously.^5^ Documented primary sites for metastasis to the submandibular gland include the breast, thyroid, lung and genitourinary organs.^3,6^ The breast is the most well-described primary site.^3,4^ To our knowledge, adenocarcinoma of the oesophagus as the primary site of metastasis has never been described in the published literature.

Conversely, metastases to the parotid gland usually derive from a local primary site and spread via the lymphatic route. Primary sites include melanomas and squamous cell carcinomas from the head and neck region, and also less frequently thyroid and tonsil primaries.^2,3^

Though both parotid and submandibular salivary glands have paraglandular lymph node networks, it has been hypothesized that the extensive intraglandular lymphatics surrounding the parotid gland render it vulnerable to local lymphatic spread. On the other hand the virtual absence of intraglandular lymphatics around the submandibular gland make it much less susceptible to such spread.^5,6^

Local lymphatic involvement is found in up to 75% of cases of oesophageal carcinoma. Haematogenous spread is also not infrequent, often affecting the lungs, liver and bones, and less commonly the adrenals and peritoneum.^7^ The sensitivity of PET scan for detection of distant oesophageal metastatic disease is estimated to be between 48% and 90%. As was the case for our patient, in up to 10–20% of cases of potentially resectable oesophageal cancer PET scan can reveal unsuspected distant metastases. PET scan should therefore be considered as an adjunct to CT scanning when staging patients.^8^

Dysphagia is traditionally investigated with upper gastrointestinal endoscopy, however, if the clinician wishes to exclude abnormalities in the oral and pharyngeal phases of swallowing, other investigations such as videofluoromanometric study may prove useful. The option of videofluoromanometric study is also particularly relevant in patients who are deemed too frail to undergo invasive endoscopic testing.^9^

In total, just over a 100 cases of metastases to the submandibular gland have been identified in the literature.^3^ With limited data available, the prognosis of submandibular metastases remains indeterminate. However outcomes are likely to be poor, reflecting the underlying disease process.

Early and accurate diagnosis of submandibular gland mass lesions is important and may have significant influence on appropriate patient care and management. Patients should be evaluated and triaged on clinical assessment, usually by a Head and Neck specialist, and then referred for imaging.

In most cases high-resolution ultrasound is the initial diagnostic technique of choice. The submandibular glands are superficial and thus amenable to ultrasound assessment.^10^ With ultrasound guidance a tissue diagnosis can be obtained by either fine needle aspiration cytology or ultrasound-guided core biopsy. There is increasing utilization of core needle biopsy in diagnosis of salivary gland lesions owing to the perceived advantages of a core of tissue over cells.^11^ A core can be sent for immunohistochemical analysis, with improved potential diagnostic rates for malignancy in particular. Ultrasound and biopsy results determine the need for further imaging and guide appropriate patient management.

## Learning points

1. Metastases to the salivary glands are infrequent. When they do occur, metastases more commonly spread to the parotid gland.
2. Metastatic spread to the submandibular gland is rare, and arises from remote infraclavicular sites via the haematogenous route. The most frequently implicated primary site is the breast.
3. Although unusual, metastatic disease should be considered in the differential for a submandibular gland mass, particularly in patients with a history of malignancy. Appropriate imaging and biopsy should facilitate diagnosis.

## Consent

Written informed consent for the case to be published (including images, case history and data) was obtained from the patient(s) for publication of this case report, including accompanying images.
